# Pharmacology of novel treatments for COPD: are fixed dose combination LABA/LAMA synergistic?

**DOI:** 10.3402/ecrj.v2.26634

**Published:** 2015-03-16

**Authors:** Domenico Spina

**Affiliations:** Sackler Institute of Pulmonary Pharmacology, Institute of Pharmaceutical Science, Pharmacology and Therapeutics, King's College London, London, UK

**Keywords:** synergy, dose equivalence, LABA, LAMA, phosphodiesterase, glucocorticosteroid, p38 MAPK

## Abstract

Bronchodilators are mainstay for the symptomatic treatment of chronic obstructive pulmonary disease (COPD) and the introduction of long-acting bronchodilators has led to an improvement in the maintenance treatment of this disease. Various clinical trials have evaluated the effects of fixed dose long-acting β_2_-agonists (LABA)/long-acting anti-muscarinics (LAMA) combinations and documented greater improvements in spirometry but such improvements do not always translate to greater improvements in symptom scores or reduction in the rates of exacerbation compared with a single component drug. An analysis of whether this significantly greater change in spirometry with combination therapy is additive or synergistic was undertaken and is the subject of this review. Bronchodilators are not disease modifiers and whilst glucocorticosteroids have been shown to reduce rates of exacerbation in moderate to severe COPD, the increase risk of pneumonia and bone fractures is a motivation enough to warrant developing novel anti-inflammatory and disease-modifying drugs and with the expectation of positive outcomes.

Chronic obstructive pulmonary disease (COPD) remains a significant disease burden that is expected to continue well into this century. Recent estimates from the World Health Organization indicate that 65 million people have moderate to severe COPD and is the cause of 5% of deaths globally and is expected to be the third leading cause of death worldwide in 2030 (http://www.who.int/respiratory/copd/en/). COPD is a largely preventable disease since cigarette smoking is a major causative factor whilst other risk factors including pollution from indoor burning of biomass fuel, outdoor pollution and occupational hazards, highlight the need for more prevention strategies.

Cigarette smoking is a major causal factor in the pathogenesis of COPD and smoking cessation is obviously an important part of COPD management, apart from other life style adjustments (1). In addition, lung volume reduction surgery may offer benefit to some patients with severe emphysema (2–4). The focus of the current review is on pharmacotherapy and clearly there is an unmet need to find new and effective pharmacological treatments that could either counter the effects of cigarette smoking, or alternatively slow the rate of progression of this disease.

Pharmacological treatments of COPD are largely palliative with bronchodilators forming a cornerstone in the management of this disease (5–7). Currently no single class or combinations of drugs modify the course of the disease and whilst glucocorticosteroids can improve lung function, quality of life and reduce exacerbations, they do not alter the long-term decline of lung function, remodelling (e.g. fibrosis of the small airways) and destruction of alveolar tissue in emphysema, features that are characteristic of the disease. Thus, no COPD medications so far can be classified as disease modifiers (5, 6). Indeed, recent studies suggest that withdrawing glucocorticosteroids in patients with severe COPD taking triple therapy does not appear to be associated with a deterioration in disease despite a significant fall in forced expiratory volume in 1 s (FEV1) (8). The introduction of the PDE4 inhibitor, roflumilast for moderate to severe COPD, whilst of benefit, has not provided a change in direction for the treatment of this disease, limited in part by bothersome gastrointestinal side effects (9, 10). As a consequence of all of the above, there is intense interest in developing novel anti-inflammatory drugs for the treatment of COPD (6).

Current treatment guidelines for pharmacological treatment of COPD consist of an algorithm that relies on indices such as respiratory symptoms, lung function and the risk of experiencing an exacerbation (11). Whilst short-acting β_2_-agonists (SABA) and anti-muscarinic drugs are often used in the milder forms of the disease, so as to provide immediate bronchodilator relief, long-acting bronchodilators have become central in the maintenance therapy of COPD. Currently there are many examples of combinations of long-acting β_2_-agonists (LABA) and anti-muscarinics (LAMA) that are used to treat COPD. Increasingly, fixed combinations of these drugs are likely to become the norm (7). Other long-acting bronchodilator drugs including mixed PDE3/4 inhibitors (12); single molecule muscarinic antagonists/β_2_-agonists (MABA) (13); and PDE4 inhibitors ‘linked’ to LABA's (14) are in development. This review will discuss whether fixed dose combinations offer an advantage of either bronchodilator alone or in combinations, and whether there is evidence for synergism. It will also briefly discuss examples of novel anti-inflammatory approaches for the pharmacological treatment of COPD.

## LABA

β_2_-Agonists reduce airflow limitation in COPD by increasing airway diameter as a consequence of a direct relaxant activity on airway smooth muscle. β_2_-adrenoceptors occur throughout the airways, principally on airway smooth muscle, but also on a variety of pulmonary cells including epithelium, submucosal glands, and mast cells. To what extent activation of β_2_-adrenoceptors on non-airway smooth muscle cells contributes to reducing airway obstruction in COPD remains to be resolved (15, 16). β_2_-Agonists can be broadly classified according to their duration of action, hence SABAs including salbutamol, terbutaline and fenoterol have pharmacodynamic half-lives between 2–6 h (15) whereas LABA's including salmeterol and formoterol require twice daily treatment (15), while ultra-LABAs, e.g. indacaterol, require once-a-day dosing (17). Other β_2_-agonists, which are currently being developed as once-a-day treatment, include vilanterol (18), olodaterol (19), carmoterol (20), abediterol (21), milveterol (22) and TD-5471 (23). The clinical effectiveness of these drugs for the treatment of COPD is not surprising given their similarities in efficacy in activating the canonical Gs protein pathway leading to elevation of cyclic AMP (24). These LABA's have similar association and dissociation kinetics for the β_2_-adrenoceptor and as a result their long duration of action is attributed to drug efficacy and/or retention within the airways and close proximity to β_2_-receptors in airway smooth muscle. Whilst the relative clinical potency of this drug class may differ, there is no demonstrable difference in clinical effectiveness, as exemplified by the different ultra-long-acting LABAs in terms of the degree of improvement in lung function (25). Their utility in the management of COPD is clear and numerous clinical studies report improvement in baseline lung function leading to a reduction in residual volume and deflation of the lung which is reflected as improvement in symptoms, quality of life and reduced incidence of exacerbations (26, 27).

## LAMA

The introduction of tiotropium bromide has proven to be beneficial for the management of COPD as shown in clinical trials in terms of improvements in respiratory symptoms, lung function (FEV1), quality of life and reduction in the frequency of exacerbations (28). As a consequence other LAMA's including glycopyrronium bromide (29), aclidinium bromide (30), umeclidinium bromide (31, 32) and dartropium bromide (33), are in clinical development. This drug class is not generally used in the treatment of asthma, although, tiotropium bromide has been shown to produce bronchodilation of a similar magnitude to salmeterol and proved clinically effective in patients with difficult to control asthma (34–36). The long duration of action of anti-muscarinic drugs has been attributed to a slower off-rate from the M3- receptors versus the M2-receptor; however, it is now recognized that these rate constants have been overestimated as a result of *in vitro* binding studies undertaken under non-physiological conditions (37). Their long duration of action has been attributed to high affinity for muscarinic receptors and to retention within the lung following inhalation (37). Similar to LABAs, clinical trials have also shown chronic use of LAMAs not only reduces airflow limitation due to the disease but are also associated with improvements in quality of life, symptom scores and reduced exacerbations. The latter most likely is due to the ability of LAMAs to suppress mucus secretion thereby reducing the colonization with bacteria that trigger exacerbation events (26, 27).

## Combination LABA/LAMA

There is increasing evidence that LABA/LAMA combinations can cause greater improvements in airflow limitation than either component drug alone (7). This might be due to suboptimal doses with either component, and hence, additional bronchodilation afforded by the combination. It has been suggested that β_2_-receptors that are located pre-junctionally on parasympathetic nerve terminals can suppress acetylcholine release thereby restricting any potential functional competition by acetylcholine at post-junctional muscarinic receptors on airway smooth muscle and submucosal glands occupied by LAMA (5, 15, 16). Post-junctional M2-receptors on airway smooth muscle are negatively coupled to adenylyl cyclase, hence, a non-selective muscarinic antagonist would inhibit a mechanism which would restrict the ability of LABAs to raise intracellular cyclic AMP in airway smooth muscle cells. Such a hypothesis is questionable given the explanation proposed to account for the long duration of action of LAMAs because of more favourable and faster off-rates from pre-junctional M2-receptors. A third possibility is that β_2_-agonists and LAMAs might act synergistically to promote bronchodilation (38, 39).

### Are LABA/LAMA combinations synergistic?

Synergy is defined as the phenomenon whereby the pharmacological response to two drugs of different classes given in combination exceeds the response that could be explained by their additive effect. Studies investigating the pharmacological effect of combinations of drugs including antimicrobials (40), chemotherapies (41) and analgesics (42, 43) showed documented evidence of synergism. This phenomenon offers numerous advantages including improvement in clinical effectiveness, reducing the incidence of drug resistance or pharmacological tolerance; and reducing the incidence of side effects of these drugs since potentially lower pharmacological doses of the component drugs can be employed. Whilst synergy is a biological (functional) effect, its evaluation requires a mathematical approach in which the observed effects of drug combinations are compared with the theoretical additive effect (or zero interaction) of the drug combination. Several methods exist to evaluate synergy including the Bliss independence model and Loewe additivity model (44, 45), the latter using an isobolographic technique for the comparison of the dose equivalent effect of drugs when used alone compared with their combined effect. The use of dose equivalence is attractive because it requires a comparison of the dose–response relationship for two drugs (though it is possible to undertake an analysis of *n* combinations of drugs) at different effect levels (e.g. between 10 and 90% Emax) to calculate the zero interaction (i.e. theoretical additive response). This can be represented by a 3D response surface that can be used to compare all possible combinations of drug pairs. Furthermore, with the aid of computing this mathematical approach is amenable to analysis and to determine statistical significance (45–48). Whilst much of our understanding of drug synergy stems from *in vitro* studies, these mathematical approaches can be used to study drug synergy in human subjects. Indeed, a number of studies have used an isobolographic method to demonstrate synergy between various combinations of anaesthetics and of analgesics in clinical studies (Table 1). A similar question as to possible synergism should be asked with the increasing move to fixed dose combinations of LABA/LAMAs for the management of COPD (7). The mathematical approach adopted in this review is described in the Appendix and a more in-depth description can be found in several review articles on this subject (45–48).

**Table 1:** Some examples of the use of a mathematical approach to investigate additivity or synergy for drug combinations in man

Drug combination	Interaction/outcome measure	Method	Reference
Remifentanil and sevofluorane	Evidence for synergism/sedation	Analysis of response surface/isobolographic	(49)
Tramadol and acetaminophen	Evidence of synergism/analgesia	Isobolographic	(50)
Tramadol and mefamizol	Evidence for synergism for some dose combinations/analgesia	Isobolographic	(51)
Clonidine and fentanyl	Evidence for additivity/post-surgical pain	Isobolographic	(52)
Neostigmine and clonidine	Evidence for additivity/analgesia	Isobolographic	(53)

#### Pre-clinical studies

A number of studies have investigated whether combinations of β_2_-agonists and muscarinic antagonists yielded synergistic bronchoprotection. For example, a synergistic interaction between tiotropium bromide and carmoterol (38) and tiotropium bromide and olodaterol (39) has been reported against airway obstruction in the guinea pig *in vivo*. In a third study, evidence was provided to support the view that combination of ipratropium bromide and salbutamol, in a dose ratio equivalent to Combivent^®^ was synergistic (55). The data presented in those studies is difficult to interpret since the authors did not use a mathematical approach based on drug equivalence to specifically analyse for additivity or synergy. In two out of three of these studies, the data was re-analysed based on the information provided (see Supplementary file for interested readers) and there was some evidence for drug synergy between a β_2_-adrenoceptor agonist and muscarinic antagonist.

The underlying mechanism of the synergism is not well understood although evidence is emerging from studies in guinea pig airways to suggest that the LAMA component of the combination might disinhibit Gi mediated suppression of calcium activated potassium channel opening (56). This would lead to hyperpolarization of the airway smooth muscle membrane and hence promote relaxation induced by activation of the canonical Gs pathway. This would not only lead to further activation of these ion channels but also other intracellular signalling pathways involved in mediating relaxation by the LABA component (56).

#### Clinical studies

A recent study has documented synergy between the bronchodilator effect of glycopyrronium bromide and indacaterol in COPD patients. The bronchodilator response to an inhaled dose of glycopyrronium (50 µg) or indacaterol (150 µg) alone and in combination was monitored over 3 h. In order to determine synergy, the bronchodilator response at each time point was expressed as a percentage of the maximum bronchodilation observed with salbutamol in the same patients, and then using the Bliss independence method to evaluate synergy (57). It appears that synergism was only observed during the rising phase of the bronchodilator response, but not at its peak.

Numerous clinical studies have reported the beneficial effects of the combined use of LABAs and LAMAs over a number of variables indicating benefit; these included improvement in trough FEV1, rates of exacerbation, dyspnoeic event as well as control of symptoms for tiotropium bromide/indacaterol (58) umeclidinium bromide/vilanterol (32), and glycopyrronium bromide/indacaterol (27, 59).

This beneficial effect, of the combination therapy versus drug component, appear to be most marked for the spirometric variables (mostly reflecting the large airways), while less evident on disease control and disease progression, a not unexpected finding in view of the fact that the drugs used are not considered disease modifiers. One would anticipate that a change in baseline FEV1 might be reflected by an increase in the diameter of the small airways resulting in lung deflation and a reduction in lung volume and consequently improvements in symptom scores and reduction in rates of exacerbation of symptoms and therefore the clinical relevance of the findings could be questioned (60). To date, there are no studies that have systematically compared the bronchodilator effectiveness of LABA or LAMA used alone or in combination on spirometric variables including FEF25-75, MFEF, impulse oscillometry or high-resolution computer tomography (HRCT) to monitor changes in small airway calibre. Assuming that small airway calibre is improved, the additional benefit achieved with the combination does not appear to be reflected in symptom scores. Alternatively, symptoms associated with COPD may be independent of FEV1 *per se* (61–64) and more sensitive indices that reflect residual lung volume, or use of forced oscillation techniques to monitor the calibre of small airways might show a better relationship between changes in spirometry and symptoms. Alternatively, bronchodilators can reduce airway wall stiffness which might also contribute to their ability to reduce dynamic hyperinflation and lung volume (65).

In theory the advantage of fixed dose combination over monotherapy would be to provide additional bronchodilation particularly if some patients are insufficiently dosed on monotherapy while combination therapy offers the opportunity of reducing the dose of each bronchodilator, but not at the expense of clinical effectiveness, while reducing the risk of adverse side effects with high dose monotherapy. Indeed, a greater degree of airway obstruction, indicative of more severe disease, reduces bronchodilator effectiveness particularly with lower doses of bronchodilator (25). Hence, maintaining high levels of bronchodilator tone with combination therapy could be advantageous.

None of the clinical trials that have demonstrated a greater degree of bronchodilation afforded by fixed combination doses over monotherapy were designed to specifically address the question of synergy. Therefore, an analysis was undertaken using the available literature to investigate whether fixed dose combinations of umeclidinium bromide/vilanterol and glycopyrronium bromide/indacaterol are synergistic. These studies where chosen because dose–response relationships for each of these bronchodilators have been published and large clinical trials in moderate to severe COPD have been undertaken which provides an adequate and relevant data set for analysis. Moreover, there is no evidence that there is anything demonstrably unique concerning the bronchodilator effectiveness of a range of LABA's (66) and LAMA's (31) and any difference between them could be reasonably attributed to the use of doses that were not clinically equi-effective.

**Fig. 1:**
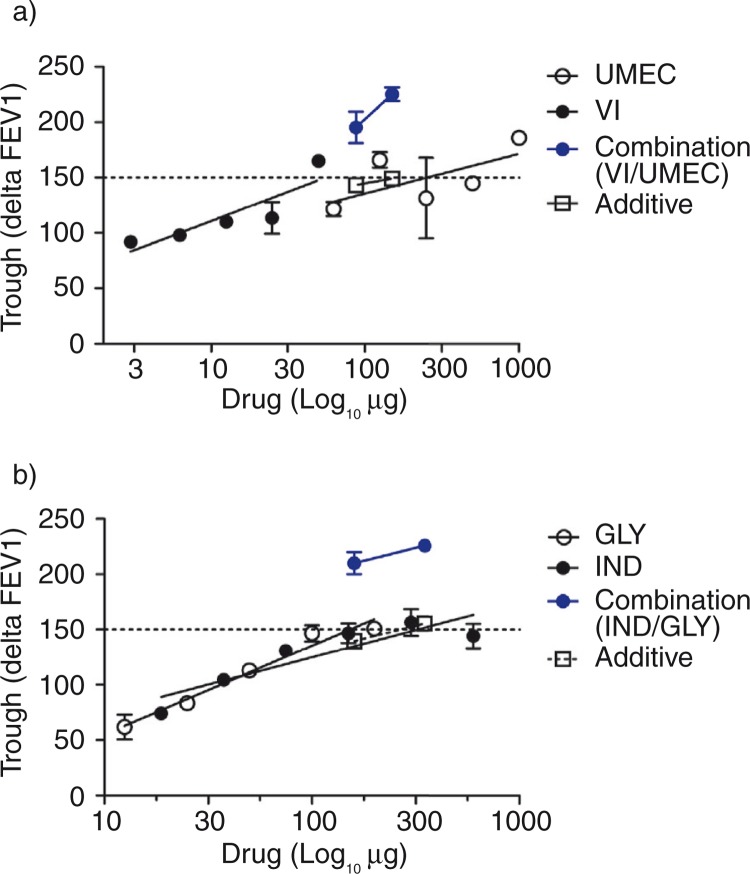
Dose–response relationships for a LABA and a LAMA alone and in a fixed dose combination LABA/LAMA in patients with moderate to severe COPD. (a) Shows a linear regression for the dose–response relationship for umeclidinium bromide (UMEC: open circles) and vilanterol (VI: closed circles) (b) glycopyrronium bromide (GLY: open circles) and indacaterol (IND: closed circles). The theoretical additive response (open squares) and the observed response (circles; blue) for fixed dose combinations of these bronchodilators are superimposed. The combination effect was shown to be synergistic (see Table 2).

Dose–response relationships for umeclidinium bromide (31, 67) and vilanterol (68) were plotted using linear regression of the log dose versus trough FEV1 after 1-month treatment. These data also included the single drug data sets from the publications that compared fixed dose combination with the either bronchodilator alone (see below). It is noticeable that the slope of the dose–response relationship is flat particularly in the case of umeclidinium bromide (Fig. 1). The dose (µg) and 95% confidence interval (95% CI) which caused a 150 mL difference in trough FEV1 was 250 (62–1010) and 55 (16–181) for umeclidinium bromide and vilanterol, respectively. A change of 160 mL in FEV1 is considered clinically important and related to a change in St George's respiratory questionnaire (SGRQ) of four units (62). Several studies have evaluated the effects of different fixed dose combinations of these agents (umeclidinium bromide/vilanterol) including 125/25 µg evaluated over a 24-week period (26, 69) and 52-week period (70) and 62.5/25 µg over a 24-week period (26, 32).

In all cases, there were significant improvements in the primary objective measure of trough FEV1 with combination versus single drugs and in other spirometric measures (e.g. peak FEV1); however, only the trough FEV1 data was analysed because dose–response relationships for these indices of symptoms was not available. As with earlier trials, the combination therapy is not always demonstrably better than single drugs in terms of risk of exacerbation rate, quality of life scores and dyspnoea scores. For example, improvement in the SGRQ score was greater with the 125/25 µg dose combination (69) compared with the single drugs, although this was not confirmed in another study in patients with similar disease severity (32). For a lower dose combination (62.5/25 µg), the improvement in trough FEV1 over the component bronchodilators did not translate to a significantly greater improvement in symptoms scores though in both studies, the risk of exacerbation was similar across all treatments (26, 32). Using a different LABA/LAMA combination, it was demonstrated that glycopyrronium bromide/indacaterol (50/110 µg) was associated with significant improvement in trough FEV1, reduced exacerbation rates and improvement in symptom scores compared with glycopyrronium bromide alone as there was no LABA arm of the trial (27). Whether some of these studies were not powered or not of sufficient duration (e.g. maximum study period was 1 year) to detect differences in rate of exacerbation and symptom score is a distinct possibility.

The theoretical additive and observed dose–response relationship for the total combined dose of umeclidinium bromide/vilanterol is illustrated in Fig. 1. The ‘bronchodilator potency’ of the combination was defined as the dose of bronchodilator which caused a 150 mL improvement in trough FEV1 and this was four times lower than that which could be ascribed to an additive effect (Table 2). Furthermore, the interaction index (alpha) was significantly different from unity which is also indicative of synergy at both dose combinations (Table 2). The latter estimate implies that a 10–20 fold reduction in the combination dose will achieve the same effector response as either drug acting alone. There was also a significant difference in the observed and the expected trough FEV1 values, again supporting the notion of synergy (Table 2).

**Table 2:** Summary of potency estimates, interaction index (alpha) and difference in effector response to assess synergy between different combinations of LABA/LAMA in moderate to severe COPD

	Parameter estimates of potency (ED50: µg)^a^	Interaction index (alpha)^b^	Delta response (observed-additive, mL)^b^
Umeclidinium	250 (31–2012)		
Vilanterol	55 (9–326)		
Combination (UMEC/VI)	38 (10–154)		
125/25 µg (150)		0.024 (0.015)*	76 (12)*
62.5/25 µg (87.5)		0.13 (0.15)**	51 (24)
Additive	161 (128–203)		
Glycopyrronium	152 (90–258)		
Indacaterol	320 (104–986)		
Combination (GLY/IND)	8.5		
50/110 µg (160)		0.074	70.2
50/300 µg (350)		0.051	70.6
Additive	351 (288–428)		

Values in parentheses (first column) indicate total dose (µg) for each dose combination.

a Values expressed as mean and 95% CI

b values expressed as mean (SD).

There was evidence of synergy for umeclidinium bromide (UMEC) and vilanterol (VI) dose combinations as implied by the five-fold difference in bronchoprotector potency (combination vs. additive) and interaction index<1 (values compared with the theoretical additive response i.e. population mean=1)

* *P=*1.06E-06

** *P*=0.009716 (unadjusted). Number of studies (*n*=3 for 87.5 µg combined dose; *n*=4 for 150 µg combined dose). There was a significant difference in observed–expected trough FEV1 measurement for the high (*P=0.0011) but not low dose combination (*P*=0.0675). Data for glycopyrronium bromide (GLY) and indacaterol (IND) also suggest synergy, based on a comparison of potency, combination index and delta response, but too few studies to undertake statistical analysis (*n*=2 for 160 µg combined dose; *n*=1 for 350 µg combined dose).

A second analysis of the bronchodilator effectiveness of fixed dose combinations of glycopyrronium bromide and indacaterol also shows evidence of synergy (Fig. 1, Table 2). Dose–response data for glycopyrronium bromide (71–73) as well as single dose studies (29, 74) plus dose–response data for indacaterol (25, 75, 76) was analysed using linear regression for trough FEV1 values versus log dose (µg; Fig. 1). The dose (mean with 95% CI) producing a 150 mL improvement in trough FEV1 was 152 (90–258) and 320 (104–986) µg for glycopyrronium bromide and indacaterol respectively. Various studies have examined the effect of fixed dose combinations of glycopyrronium bromide and indacaterol of 50/110 µg (77, 78) and 50/300 µg (79) and the improvement in trough FEV1 by each bronchodilator alone was also used in the determination of bronchodilator potency (Fig. 1). The ‘bronchodilator potency’ of the combination when defined as the dose which caused a 150 mL improvement in trough FEV1 was an order of magnitude greater than that which could be ascribed to an additive effect (Table 2). Furthermore, the interaction index (alpha) was less than unity again indicative of synergy at both dose combinations tested (Table 2). The analysis implies that a 10–20 fold reduction in the combination dose will achieve the same effector response as if either drug was acting alone. There was also a significant difference in the observed and expected trough FEV1 values supporting the presence of synergy. Statistical analysis of such data could not be undertaken because of the paucity of published studies of fixed dose combinations of glycopyrronium bromide and indacaterol.

It is important to acknowledge some limitations in the foregoing analysis. The data was obtained from a number of clinical studies with different treatment durations and, whilst the subjects tended to be predominantly within the moderate to severe disease classification, one cannot rule out possible differences in bronchodilator response in different patient cohorts, and differences in measurement of FEV1 between different clinical sites. The bronchodilator response to each single component in the combination clinical trials was included in the analysis to obtain better estimates of drug potency across a number of studies. However, there may have been an overestimation of the bronchodilator potency of the fixed combination and the interaction index because the dose–response relationships of each bronchodilator was characterized by low slope values and coupled with the constraint of limited number of different fixed dose combinations available for analysis. The analysis would have benefited if the fixed dose combinations had the same proportions of LABA/LAMA, and if the number of fixed dose combinations was greater than that which was available for analysis so as to give better estimates of potency and the interaction index for the combination therapy. Furthermore, each bronchodilator and different combinations of the bronchodilators should be evaluated in the same patient using crossover designs, or alternatively by recruiting a relatively large patient group and using a parallel design.

### Is bronchodilator synergy clinically relevant?

Notwithstanding these issues, fixed combinations of LABA/LAMA appeared to show a greater degree of improvement in trough FEV1 when compared with the respective single components. Analysis of synergism on clinical indices including quality of life, exacerbation rates and disease progression was not possible because dose–response relationships for the single component drugs were not available but carefully designed and sufficiently powered studies could help evaluate these missing important efficacy data. Therefore, whilst FEV1 may be a relatively poor predicator of improvements in symptom scores, either the dose–response relationship for these phenomenon are different, or more sensitive measures of small airway calibre (e.g. forced oscillatory techniques) might offer greater predictability. It is therefore likely that the combination therapy provides a complimentary coverage of airway smooth muscle relaxation with a suppression of mucus secretions which benefits the patient provided this is not at the expense of more adverse effects (60). It remains to be seen whether any purported synergy would allow a dose reduction of both component drugs whilst still affording clinical meaningful bronchodilation over a 24 h period.

## Bifunctional molecules

Another approach to achieving better drug therapy of COPD could be in the development of dual acting MABAs suitable for once-a-day treatment as exemplified by GSK961081. This drug offers the advantage of a single molecule with a single pharmacokinetic profile and potential benefits concerning formulation of one as opposed to two separate molecules which offers greater simplicity for patients undergoing triple therapy with combination LABA/LAMA/inhaled corticosteroids in COPD (13). In light of the preceding discussion concerning the potential synergistic interaction between a LABA and LAMA, it might not be unreasonable to suggest that MABA's are inherently synergistic in terms of their pharmacological effect on airway calibre. A change from baseline trough FEV1 was 215 and 277 mL with a once daily dose of 400 and 800 µg GSK961081 (13), respectively. A change that was of a similar magnitude to fixed dose combinations of LABA/LAMA was shown in this analysis to be synergistic.

**Fig. 2:**
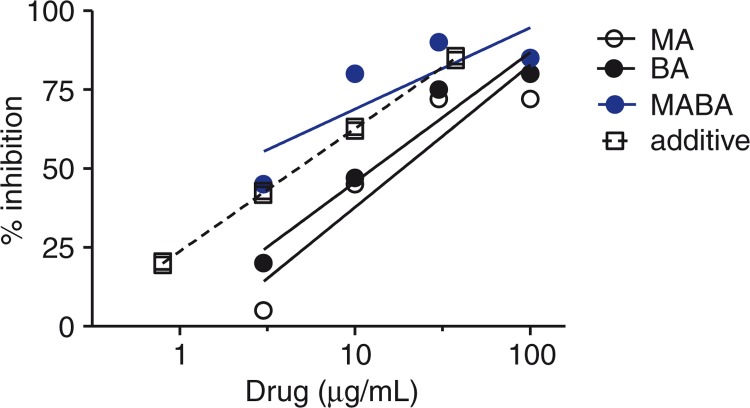
A re-analysis of the data presented by McNamara et al. (55) using a mathematical approach to evaluate synergism of the bronchoprotective effect of a MABA in anaesthetized guinea pigs. Linear regression was used to fit the cumulative dose–response curve for MABA in animals in which airway obstruction was induced by histamine to detect the β_2_-agonist (BA) component of the MABA (closed circles); or MABA in animals treated with propranolol and airway obstruction induced by acetylcholine to detect the muscarinic antagonist (MA) component of the MABA (open circles) and response to MABA (blue circles) in animals in whom airway obstruction was induced by acetylcholine. The theoretical additive line (broken lines) for each dose of MABA was calculated assuming the proportion of each component was 1:1. The observed response obtained for the MABA is shown in blue.

GSK961081 has both a β_2_-adrenoceptor agonist (carbostyryl group) and muscarinic antagonist (biphenyl carbamic acid) pharmacophore that are covalently linked. The pharmacological characteristics of this molecule include non-selectivity for different muscarinic receptor subtypes, and selectivity for β_2_- versus β_1_-adrenoceptors. This drug class is characterized by a relatively short half-life on either receptor which cannot account for the long duration of action seen *in vivo* as a bronchodilator. Such a long duration is likely due to retention of the drug within the lung environment. This is reflected in a 2–3 fold order of magnitude difference in selectivity for the airways over extra-pulmonary sites containing muscarinic and β_2_-adrenoceptors (80). One characteristic not been reported for GSK961081, but is a feature of this drug class, is the simultaneous binding to orthosteric and allosteric sites of the muscarinic and β_2_-adrenoceptor. As a consequence, these molecules can retard the dissociation of an orthosteric ligand from these receptors as exemplified by the prototypical MABA, THRX-198321 which contains a nine carbon aliphatic chain between the two binding moieties (81). This unique property might manifest in greater clinical effectiveness because of synergistic effects as suggested for THRX-200495 which contains a propyl ethyl biphenyl ether linker group (55). Whilst THRX-200495 was investigated for additivity and synergy in guinea-pigs, a formal mathematical assessment of dose equivalence was not undertaken. Using the approach described earlier (see Appendix), it would appear that this agent does demonstrate synergy, but only at low to moderate dose combinations (Fig. 2, Table 3). No formal statistical analysis could be undertaken hence the estimates are qualitative in nature. Notwithstanding the fact that the selectivity of the β_2_-agonist and muscarinic antagonist components of the MABA was assumed to be 1:1, the analysis suggests that a three-fold lower dose of the MABA is required to produce an equi-effective response with either component acting alone (Fig. 2, Table 3).

**Table 3:** Summary of potency estimates, interaction index (alpha) and difference in effector response to assess synergy for different doses of THRX-200495 against spasmogen-induced bronchoconstriction in guinea pigs

	Parameter estimates of potency (ED50: µg/mL)^a^	Parameter estimates of potency (ED50: µg/mL) (55)^b^	Interaction index (alpha)^c^	Delta response (observed–additive, % inhibition)^c^
β-Agonist	12.7 (4–38)	11.2		
Muscarinic antagonist	18.7 (3–102)	11.4		
MABA^d^	2 (0.008–419)	3.5		
1.5/1.5 (3)			0.30	25
5/5 (10)			0.16	37
15/15 (30)			0.27	27
50/50 (100)			1.189	0
Additive	4.7 (4.6–4.9)	ND		

Values in parentheses (first column) indicate total dose (µg/mL) for each dose combination.

a Parameter estimates of potency for bronchoprotection 1.5 h following drug exposure and expressed as mean and 95% CI.

b Values expressed as mean.

Bronchoconstriction was induced by histamine to measure β_2_-agonist effect of THRX-200495 (‘β-agonist’) or to acetylcholine in the presence of propranolol to measure the muscarinic antagonist effect of THRX-200495 (55). MABA refers to the effect of THRX-200495 alone. Inhibition of airway obstruction was measured 1.5 h following aerosol exposure to the bronchodilators.

c Calculation of interaction index (alpha) and delta difference between observed and additive response both based on the method of dose equivalence (see Box 1). Values expressed as mean. No statistical analysis was possible but the analysis suggests evidence for synergy at low to moderate doses of MABA compared with the additive response. There was a small increase in bronchoprotection potency (approximately two fold) when MABA is compared with the additive potency value, alpha<1 for low and medium doses of MABA and the difference in bronchoprotection between observed and additive response was approximately 30%. ND: not determined.

d ^a,c^Analysis based on the assumption that the dose of MABA can be considered as a combination of muscarinic antagonist and β_2_-agonist in a ratio of 1:1.

### Bifunctional PDE inhibitors

RPL554 is a mixed PDE3/4 inhibitor that has been demonstrated to have bronchodilator and bronchoprotective activity in mild asthmatic subjects and patients with COPD (12), a feature not observed to any degree with oral or inhaled PDE4 inhibitors. It was previously shown that cilomilast does not cause bronchodilation *per se* when measured after a single oral dose (82) and for roflumilast, the changes in baseline FEV1 in COPD develops over a period of weeks (9, 10). This lack of a direct effect of roflumilast on baseline airway tone is consistent with the lack of relaxant effect observed to this drug *in vitro* in isolated tracheal rings from guinea pigs (83, 84). Such studies demonstrate a clear distinction between RPL554 and a PDE4 inhibitor on airway smooth muscle contractility. None of the clinical trials with inhaled PDE4 inhibitors report a direct bronchodilator activity (85).

The modest success of the PDE4 inhibitor roflumilast for the treatment of COPD has kept interest in the PDE field for the development of new drugs for the treatment of this disease (9, 10). The additional bronchodilator benefit observed in patients who were maintained on LABA or LAMA is unlikely to be attributed to a direct action on airway smooth muscle, since functional studies *in vitro* demonstrate that roflumilast at concentrations that are two to three orders of magnitude greater than the Ki for inhibition of PDE4 is without demonstrable relaxant activity. Hence, the improvement in FEV1 is likely to be due to an anti-inflammatory activity as evident by the ability of this drug class to suppress neutrophil recruitment to the airways and various inflammatory biomarkers of relevance to COPD (86, 87). This is also consistent with the ability of roflumilast to cause improvements in quality of life scores and reduce rates of exacerbation by virtue of an anti-inflammatory activity. Intriguingly, a number of inhaled PDE4 inhibitors administered daily for between 1 and 6 weeks have proved disappointing in a number of phase II clinical trials (85, 88, 89) despite evidence for PDE4 inhibitory activity, however, this was not sufficient to result in any clinical benefit (89). The reason for a lack of clinical effectiveness of these highly potent and long lived inhaled PDE4 inhibitors might be a result of the presence of other PDE subtypes within the lung (e.g., PDE2, 3, 7) that might contribute to airway inflammation in COPD. The mixed PDE3/4 inhibitor, RPL554 was evaluated in a number of phase II clinical trials in both asthma and COPD subjects and shown to be an effective bronchodilator of comparable effectiveness to salbutamol, and with long duration of action following single nebulized dose (circa 6–10 h). Of particular interest was the ability of this inhaled drug, administered daily for up to 1 week, to inhibit neutrophil recruitment to the airways and consequently the first demonstration of an inhaled PDE inhibitor with an anti-inflammatory signal (12). Furthermore, relaxation of human airways *in vitro* was augmented when combinations of RPL554 and atropine or glycopyrronium bromide, was used and there was evidence of synergy using the method of dose equivalence (90).

Another strategy has been the linking of a PDE4 inhibitor with a β_2_-agonist (indacaterol) with a view to develop a bifunctional bronchodilator and anti-inflammatory drug (14). GS-5759 inhibited cytokine release, oxygen radical production and chemokine release from various inflammatory cells and it appears the β_2_-agonist component of the molecule participates in the anti-inflammatory activity of the PDE4 component. Interestingly, this bifunctional molecule was more effective than roflumilast in some of the *in vitro* assays and suggests that anti-inflammatory activity can be boosted by agents which elevate cyclic AMP within target cells (14). Hence, bifunctional or mixed PDE inhibitors offer the advantage of providing both a bronchodilator and anti-inflammatory activity which would be beneficial to the patient.

### Anti-inflammatory drugs: existing and new approaches

Like many inflammatory diseases, the complex interplay between inflammatory cells and structural cells within the lung and the mediators they release provides a wealth of potentially novel targets to treat respiratory conditions such as COPD (6). Glucocorticosteroids are potent anti-inflammatory drugs and can reduce the rate of moderate to severe exacerbation but at the expense of the development of pneumonia and fractures (91, 92) and whilst combination LABA/glucocorticosteroid was no better than a LAMA in reducing the rate of exacerbation in COPD, mortality was significantly lower and quality of life better with dual therapy (93). Withdrawal of glucocorticosteroid treatment from a triple therapy regimen did not appear to lead to a deterioration of disease but was associated with a worsening in baseline spirometry compared with placebo (8). Finally, glucocorticosteroids do not appear to reduce the annual rate of decline in FEV1 in COPD (94, 95) and patients with severe COPD do not appear to benefit in terms of reducing rates of exacerbation, from adding glucocorticosteroid to LABA compared with LABA alone despite improvement in FEV1 (96). These studies clearly highlight the unmet need to develop new types of anti-COPD agents.

The documented presence of cells of the innate and adaptive immune system in COPD could provide suitable targets (97). The proteinase hypothesis of COPD also provides numerous drug targets, for example neutrophils which are implicated in the pathogenesis of COPD, secrete neutrophil elastase which plays a role in stimulating mucus secretion and damage to the parenchymal tissue (98). Unfortunately, the neutrophil elastase inhibitor, AZD9668 was without clinical benefit in symptomatic COPD patients taking tiotropium bromide following a 3 months treatment protocol (99). The lack of effect of this treatment on biomarkers of matrix degradation indicates that pharmacodynamic relevant concentrations were not achieved in the lung and hence the primary outcome measure was not evident. Alternatively, other proteinases (e.g. MMP's) implicated in COPD would be unaffected by this treatment (98).

Targeting signalling pathways might be another approach, and in this regard many cytokines implicated in COPD signal via p38 mitogen-activated protein kinase pathways and small molecule inhibitors of this protein might prove beneficial. A relatively short 6 weeks trial with a selective p38 MAPK inhibitor, PH-797804, was associated with significant improvement in trough FEV1 of 85 mL and 92 mL for the 3 and 6 mg dose, although 120 mL is considered to be clinically relevant. Changes in dyspnoea scores were significant and deemed clinically relevant (100). The anti-inflammatory activity of PDE inhibitors has been mentioned previously and will not be discussed any further.

## Conclusion

Ultra-long-acting bronchodilators demonstrably improve measures of lung function, symptoms and reduce rates of exacerbation and therefore are used in the maintenance therapy of COPD. Fixed dose combinations will increasingly be used in the management of moderate to severe COPD, and clinical trials suggest that improvements in lung function are significantly greater than with either monocomponent alone. An analysis of the clinical data indicated synergism for bronchodilation. However, this should be confirmed with appropriately designed clinical trials. So far, the synergistic benefit does not appear to translate into improvements in symptom scores and exacerbation rates. Neither is it clear if synergic activity improves small airway function or induces disease-modifying effects. Novel bronchodilator agents that combine both bronchodilator and anti-inflammatory activity offer a new type of treatment modality for COPD patients as the field awaits news of positive clinical trials with molecules which specifically target the inflammatory response, and document superiority over glucocorticosteroids and roflumilast.

## Supplementary Material

Pharmacology of novel treatments for COPD: are fixed dose combination LABA/LAMA synergistic?

Pharmacology of novel treatments for COPD: are fixed dose combination LABA/LAMA synergistic?

Pharmacology of novel treatments for COPD: are fixed dose combination LABA/LAMA synergistic?

Pharmacology of novel treatments for COPD: are fixed dose combination LABA/LAMA synergistic?

Pharmacology of novel treatments for COPD: are fixed dose combination LABA/LAMA synergistic?

## Figures and Tables

**Fig. A1:**
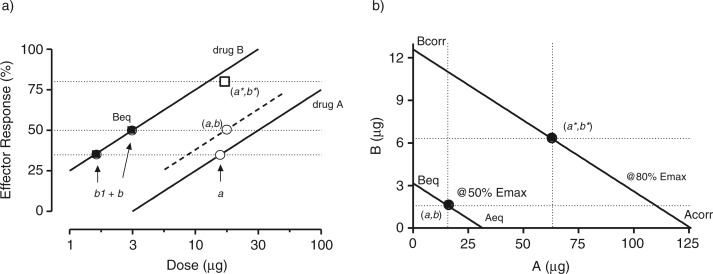
Diagrammatic representation of the mathematical approach used to calculate synergy (45, 46, 48). (Panel a) The ordinate represents the response (% Emax) and the abscissa represents log dose. In this example, assume the dose–response relationship for two drugs, A and B was fitted by linear regression and gave rise to the following relationships YA=50.Log_10_[A] – 25 (EC50A=31.6 µg) and YB=50.Log_10_[B]+25 (EC50B=3.16 µg). In this example, the dose ratio (R) is 10. In this example, assume a drug pair (*a, b*) was to be evaluated for synergy (15.8, 1.58 µg) and yielded an effector response of 50% Emax (panel a, open circle, angle dotted line). (Panel b) Linear isoboles representing the zero interaction for dose pairs which give rise to an effector response of 50% and 80%. The *a, b* pairing (15.8, 1.58 µg) lies on the zero interactive isobole (@50 % Emax). If this pairing gave rise to an effector response of 80%, this would be synergistic (*a, b* lies beneath the 80% linear isobole). See text for explanation for the calculation of the interaction index, alpha.

## Appendix

A number of mathematical approaches are available to assess the additive or synergistic interaction for drug combinations and are based on dose equivalence (45–48). Other methods include the Bliss independence method although this method is not consistent with the mathematical approach taken in this review (48).

For two drugs used in combination, then the following intercept equation is used to determine zero interaction or additive effects:

$$\frac{a}{A}+\frac{b}{B}=1$$

In this relationship, the numerator terms *a* and *b* represent the concentration (or dose) pairs, usually in a constant proportion, that, in combination, produce a biological response (e.g. 50% Emax). Consequently, there will be an equivalent concentration of either drug *alone* (denoted A, and B, respectively) that will cause the same biological response as this *a, b* pair (see Fig. A1). If a drug combination produces an additive effect then the sum of these ratios is unity, whilst values less than unity are indicative of synergy whilst values>1 are indicative of antagonism.

The dose–response relationship for drug A *alone* (or drug B *alone*) can be described by a logistic equation of the form:

$$\frac{E_{A}}{E_{M}}=\frac{{\left[A\right]}^{n}}{{\left[A\right]}^{n}+{\left[EC50_{A}\right]}^{n}}$$

Where E_A_ is response to dose, E_M_ is the maximum response (Emax), E_A_/E_M_ represents the fractional effect (0–1), EC50_A_ is the effective concentration for a 50% response, A the dose under consideration and *n* the Hill slope coefficient.

Alternatively, one could use linear regression analysis to fit (within limits) the log concentration response versus effect relationship which is particularly useful when studying drug activity *in vivo* and can be legitimately used to describe the cumulative dose–response curve between 5 and 95% of the maximal response or effect E_M_. This approach has been used for the analysis of synergy presented in this paper.

The following linear functions describe the concentration–response relationship for drug A and drug B, respectively:

$$Y_{A}=\text{intercep}{\text{t}}_{A}+m_{A}\text{x}Log[A]$$

$$Y_{B}=\text{intercep}{\text{t}}_{B}+m_{B}\text{x}Log[B]$$

Where Y represents the effector response for drug A or drug B with the corresponding intercept and slope (*m*) for drug A and B. An illustration of this relationship is shown in Fig. A1 (panel a). Drug pairs are usually chosen in the same proportions such that dose equivalence can be ascribed between the *a,b* dose pairs. If Y_A_ and Y_B_ are parallel, the dose ratio (R=A/B) can be calculated and for each dose pair, the intercept equation rearranged as follows:

$$\frac{a}{R}+b=B_{eq}\;OR\;a+R.b=A_{eq}$$

Where, B_eq_ is the concentration of drug B *alone* that is equi-effective (or equivalent) to the combination pair (*a, b*). Alternatively, this could be expressed as a function of A_eq_ if the dose equivalence of A was desired.

For any concentration (or dose) of *a* (in a dose pair) that yields a particular effector response, the equivalent dose, *b1* (=a/R if the two lines are parallel) is then added to *b* (from the dose pair) to give the concentration of drug B *alone* that would yield a similar response to the *a, b* pair (i.e. *b1, b*) pairing (Fig. A1, panel a). Consequently, B_eq_ represents the dose of drug B acting alone that is equivalent to a particular dose pair (*a, b*) and yield a similar effector response (see Fig. A1, panel b). One can calculate a theoretical line of additivity (zero interaction) from any number of dose pairs (within the limits discussed above) used in an experiment (Fig. A1, panel a, angle dotted line). Consequently, the additive EC50 value (with confidence interval) can be compared with the observed EC50 of the response to the drug pairs (with confidence interval).

In many instances, the dose ratio between drug A and drug B is not constant and under these circumstances, the simultaneous solution of Y_A_=Y_B_ and the intercept equation is required to calculate the theoretical line of additivity. In this instance *b1* is captured by re-arrangement of Y_A_=Y_B_ and for simplicity:

$$b1=\frac{\text{intercep}{\text{t}}_{A}+m_{A}\text{x}Log[A]}{m_{B}}-\text{intercep}{\text{t}}_{B}$$

One can calculate a theoretical line of additivity from any number of dose pairs used in an experiment (Fig. A1, panel a, angled dotted line) by calculating B_eq_ (=b+b1) and insertion into the Y_B_ function (or A_eq_ into the Y_A_ function). Consequently the additive EC50 value (with confidence interval) can be compared with the actual EC50 (with confidence interval) observed from the combination concentration–response relationship. One could then calculate whether these potency values were statistically different by using a single sample *t*-test, where the population mean in this case is the EC50 value derived from the line of additivity.

In a final step, for any observed effector response (a*,b*) to a particular drug combination (e.g. 80% Emax, Fig. A1) one can calculate the corresponding concentration of drug A alone (Acorr) or drug B alone (Bcorr) that would yield the same effector response, by re-arranging and solving the linear regression curve for each drug respectively. The ratio A_eq_/A*corr* or B_eq_/B*corr* gives rise to the interaction index (alpha) (48) or using a different method of dose equivalence, one can calculate the combination index (CI) (54). Values<1,=1 and>1 represent synergy, additivity and antagonism respectively.

Graphpad prism was used to undertake linear regression of the component log dose versus response relationship to obtain estimates of intercept and slope and for graphical presentation. The calculation of the line of additivity and interaction index was undertaken using SAS (version 9.3) by solving the simultaneous set of linear equations described earlier (i.e. Y_A_, Y_B_ and the intercept equation).

As an illustration, for any dose pair (*a, b*), there will be an equivalent dose (*b1=a*/R) for *a*. The effector response of this combination (*b1* and b) will be equivalent to drug B acting *alone* (i.e. B_eq_). In this example, the dose equivalent (i.e. *b1*) for drug *a* in this combination (a=15.8 µg) is 1.58 µg (=a/R). Hence, B_eq_ is the sum of the dose equivalent of *a* (*b1*) and the dose of *b* in this *a, b* pair (i.e. 1.58+1.58=3.16 µg) (panel a). Insertion of this dose into the Y_B_ relationship gives an effector response of 50% (dotted horizontal line). Conversely, using a similar approach one could calculate A_eq_ (=31.6 µg) and insertion of this value into the Y_A_ relationship would also yield an effector response of 50% Emax (not shown in panel a).

In summary, the total combination dose of this *a, b* pair (i.e. 17.38 µg) should give an effector response of 50% if the relationship is additive (*a, b* at 50% Emax, angled dotted line). We could calculate the interaction index (alpha) with the aid of the intercept equation, and conclude that the interaction for this particular drug combination is additive (i.e. 1.58/3.16+15.8/31.6=1). If multiple combinations of the two drugs in the same proportions are used then the line of additivity (dotted angled line) can be compared with the observed combination dose–response relationship (not shown). For example, one could compare the EC50 values for the theoretical additive curve and the observed combination curve.

In the Bliss independence method, the relationship E(a, b)=Ea+Eb – (Ea×Eb), where E represents the fractional response (between 0 and 1) and *a* and *b* represent the concentration (or dose) within each combination pair. In this example, the effector response for *a* (15.8 µg) and *b* (1.58 µg) *alone* is 0.35 (or 35% Emax), Hence, E(a, b)=0.58 (or 58%). This value is greater than 0.5 (or 50%). A statistical test would be required to ascertain whether this value was different from the 50% value, but illustrates the lack of compatibility with the intercept model.

Let us now assume the observed response for this combination was 80% Emax (open square, panel a; filled circle @80% isobole panel b). We can now calculate the corresponding dose for drug A (Acorr=125.9 µg, by re-arrangement and solving Y_A_) and for drug B (Bcorr=12.59 µg, by re-arrangement and solving Y_B_) that gives the same effector response (=80% Emax) if either drug was used *alone*. Consequently, the interaction index (alpha) can be represented as either the ratio A_eq_/Acorr (=31.6/125.9=0.25) or B_eq_/Bcorr (3.16/12.59=0.25) or a/Acorr+b/Bcorr (=15.8/125.9+1.58/12.59=0.125+0.125=0.25) and this holds true if the lines are parallel. This approach of dose equivalence can be extended if logistic equations are used for drugs with different maximal effects, non-constant dose ratio and differ in Hill slope. Using the Bliss independence method, then E(a, b)=0.35 (=35% Emax) which is less than the observed response (i.e. 0.8=80% Emax) to the combination and would be considered a synergistic interaction.

One can also generate isoboles to diagrammatically represent relationships that are additive and synergistic as defined by the intercept relationship (a/A+b/B) at different effector levels and for all possible combinations (within limits, Figure A1 panel b). Those pairings that lie below the isobole are considered synergistic, those on the line additive and above the line, antagonism. In this example, the interaction (alpha) of this pairing (*a, b*) would have a value of unity (i.e. 15.8/31.6+1.58/3.16), if it were additive and therefore lie on the additive isobole. If the *a, b* pair resulted in an effector response 80% Emax then an alpha value of 0.25 denoting synergy as it lies below the Acorr, Bcorr line of additivity for the 80% effector response. This value of alpha would indicate that a four-fold reduction in the combination dose would achieve the same effector response as either drug acting alone. Finally, extrapolating from the zero axis through the (a, b) pair will intersect the 80% Emax intercept (*a*, b**: 63.2, 6.32 µg, respectively), a dose combination that would now lie on the additive curve for this response level.
