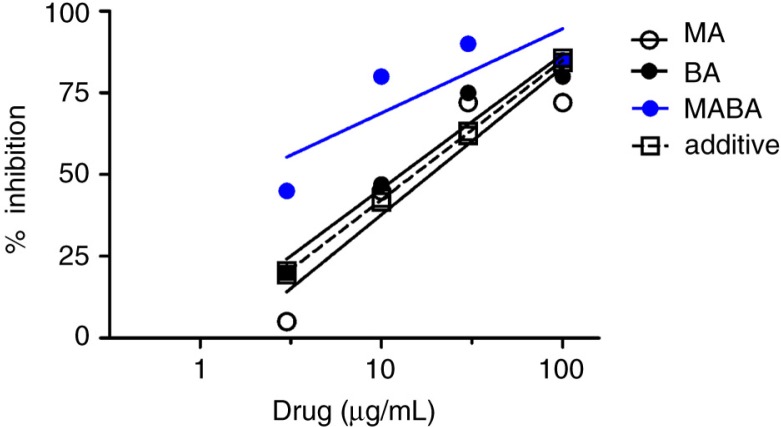# ECRJ Corrigendum

**DOI:** 10.3402/ecrj.v2.28021

**Published:** 2015-04-17

Published in *European Clinical Respiratory Journal* 16 March 2015. Citation: European Clinical Respiratory Journal 2015, **2**: 26634 - http://dx.doi.org/10.3402/ecrj.v2.26634

Following publication of this article the author noted an error in Figure 2. The additive line (dotted line) was positioned incorrectly. This has now been corrected in the revised figure. Consequently, the parameter estimate of potency (ED50: µg/mL) for the additive line is also corrected to 15.2 (14.7–15.6). This change does not alter the value of the calculated interaction index (alpha) or the delta response (observed-additive, % inhibition) values in Table 3.

**Fig. 2:**