# Supplementary figures and images for: Pharmacology of novel treatments for COPD: are fixed dose combination LABA/LAMA synergistic?
Source: Eur Clin Respir J. 2015 Mar 16;2:10.3402/ecrj.v2.26634. doi: 10.3402/ecrj.v2.26634 (PMC4629759; doi:10.3402/ecrj.v2.26634)

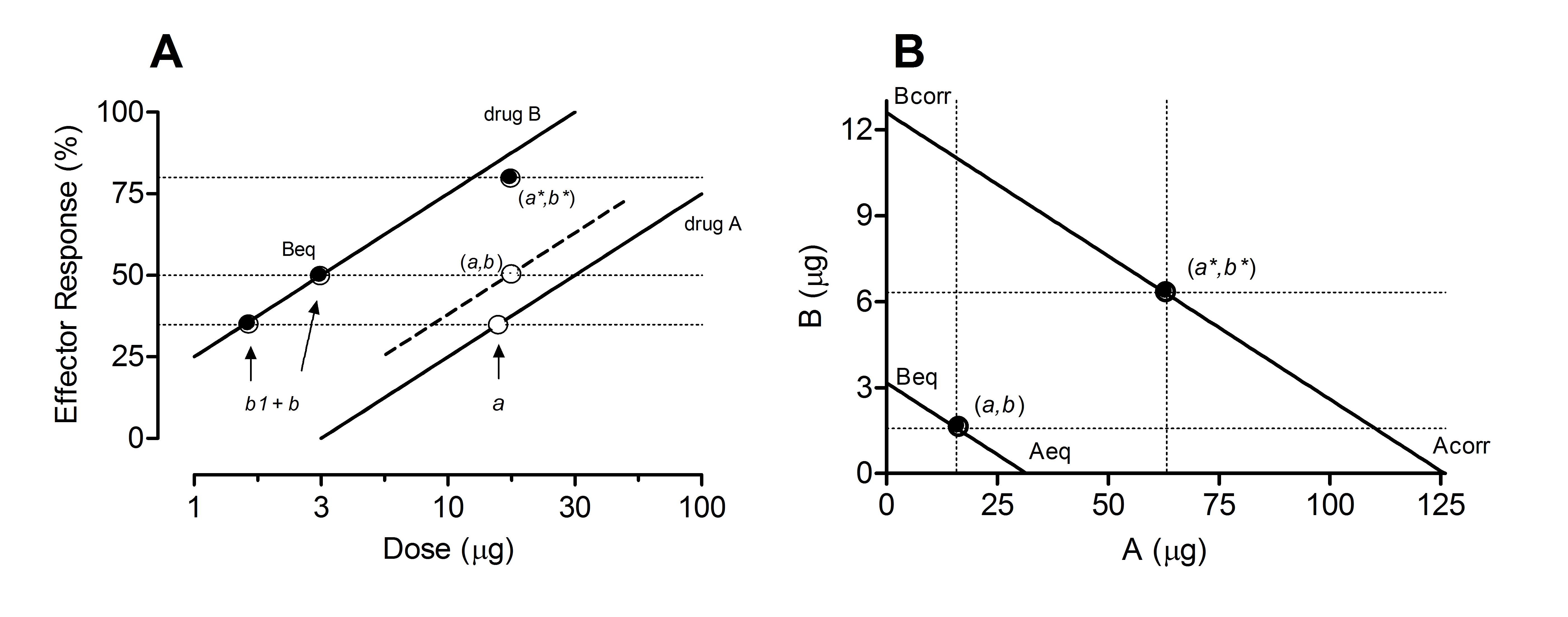

Supplement: Pharmacology of novel treatments for COPD: are fixed dose combination LABA/LAMA synergistic? [file ECRJ-2-26634-s001.jpg]

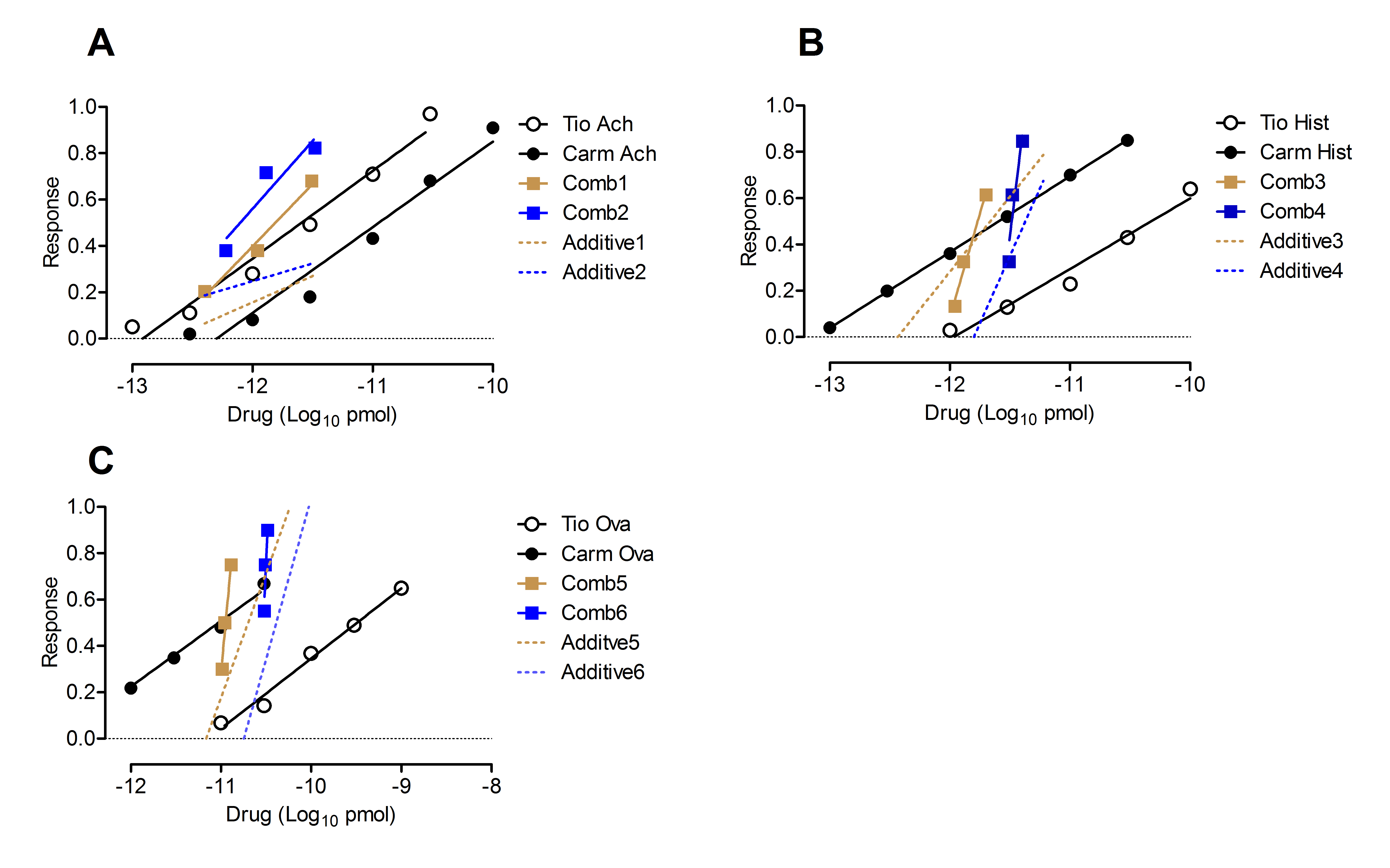

Supplement: Pharmacology of novel treatments for COPD: are fixed dose combination LABA/LAMA synergistic? [file ECRJ-2-26634-s002.jpg]

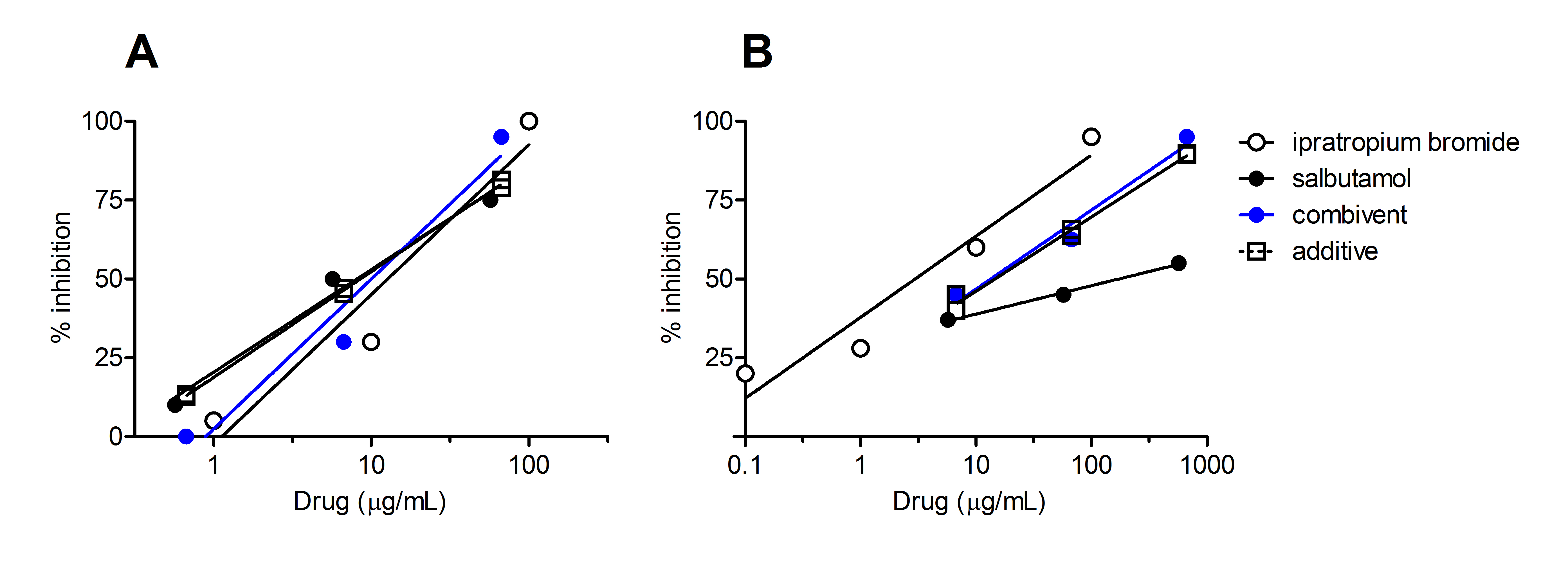

Supplement: Pharmacology of novel treatments for COPD: are fixed dose combination LABA/LAMA synergistic? [file ECRJ-2-26634-s003.jpg]

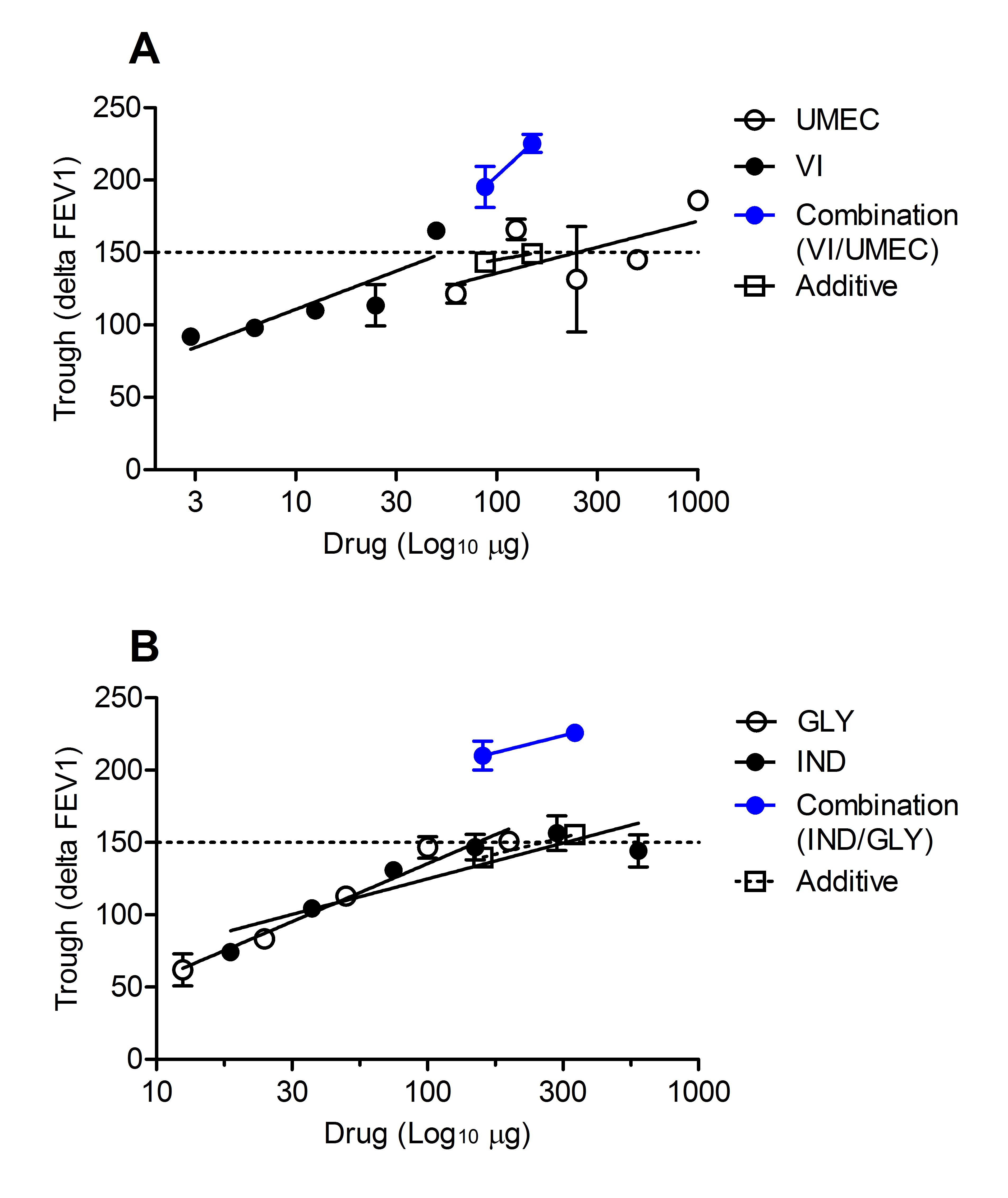

Supplement: Pharmacology of novel treatments for COPD: are fixed dose combination LABA/LAMA synergistic? [file ECRJ-2-26634-s004.jpg]

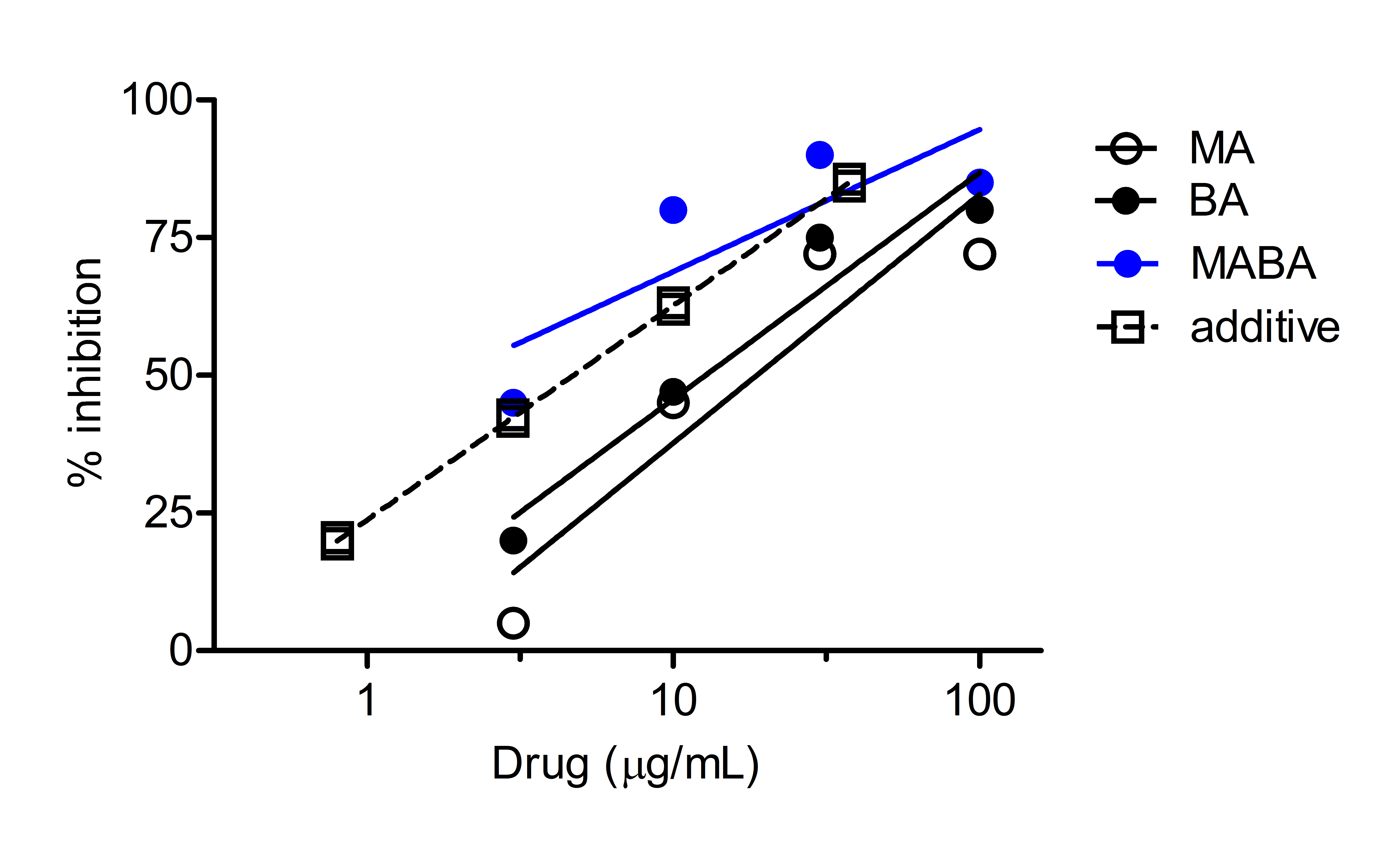

Supplement: Pharmacology of novel treatments for COPD: are fixed dose combination LABA/LAMA synergistic? [file ECRJ-2-26634-s005.jpg]
